# Deep Mutational Scanning in Immunology: Techniques and Applications

**DOI:** 10.3390/pathogens14101027

**Published:** 2025-10-10

**Authors:** Chengwei Shao, Siyue Jia, Yue Li, Jingxin Li

**Affiliations:** 1 School of Public Health, Southeast University, Nanjing 210009, China; 230239097@seu.edu.cn; 2 Jiangsu Provincial Medical Innovation Center, National Health Commission Key Laboratory of Enteric Pathogenic Microbiology, Jiangsu Provincial Center for Disease Control and Prevention, Nanjing 210009, China; jiasiyue0616@sina.com; 3 Nanjing Vazyme Biotech Co., Ltd., Nanjing 210033, China; liyue@vazyme.com

**Keywords:** immunology, mutation, sequencing, antibody

## Abstract

Mutations may cause changes in the structure and function of immune-related proteins, thereby affecting the operation of the immune system. Deep mutational scanning combines saturation mutagenesis, functional selection, and high-throughput sequencing to evaluate the effects of mutations on a large scale and with high resolution. By systematically and comprehensively analyzing the impact of mutations on the functions of immune-related proteins, the immune response mechanism can be better understood. However, each stage in deep mutation scanning has its limits, and the approach remains constrained in several ways. These include data and selection biases that affect the robustness of effect estimates, insufficient library coverage and editability leading to uneven representation of sites and alleles, system-induced biased signals that deviate phenotypes from their true physiological state, and imperfect models and statistical processing that limit extrapolation capabilities. Therefore, this technology still needs further development. Herein, we summarize the principles and methods of deep mutational scanning and discuss its application in immunological research. The aim is to provide insights into the broader application prospects of deep mutational scanning technology in immunology.

## 1. Introduction

In recent years, advances in gene synthesis, gene editing, and high-throughput sequencing, together with continual improvements in bioinformatics, have markedly expanded the use of deep mutational scanning (DMS, also known as saturation mutation screening) in biomedicine [1,2,3]. DMS is a technology that combines deep sequencing with gene or genome libraries generated by programmed allelic mutations, thereby linking genotypes with phenotypes through high-throughput platforms to detect the functional effects of mutations at each single nucleotide position in a gene or genome [4]. Because genetic variants frequently alter amino acid sequences and thus protein structure and function, DMS has become a powerful approach for dissecting sequence–function relationships. At scale, it quantifies the impact of genetic variation efficiently and at comparatively low cost [5].

The normal function of the immune system depends on the coordinated activity of multiple proteins, including but not limited to antibodies, cell surface receptors, a part of signal transduction molecules, and effector molecules, which play a key role in identifying, responding to, and clearing pathogens and other foreign substances. The structure and function of these proteins may be affected by their own gene mutations, which may lead to enhanced, diminished, or dysregulated immune function [6,7]. Therefore, DMS holds substantial potential and broad prospects in immunology. Its key strength lies in the ability to link “site–variant–function” relationships in high-throughput platforms, thereby mapping molecular-level site effects onto immune outcomes at the cellular and even organismal levels. Compared with traditional mutational analysis, DMS affords broader coverage and higher resolution for assessing the consequences of variation [5]. Systematic, high-throughput characterization of immune-related gene variants is expected to illuminate mutation-driven mechanisms of immune function, refine our understanding of immune responses, and inform the development of immunotherapeutics, vaccines, and immune modulators.

This review describes the basic principles and methods of DMS and introduces the widely utilized technical frameworks at each step of the DMS process. It compares the advantages, limitations, and appropriate use cases of DMS in studies of antibody, antigen, and T-cell receptor (TCR), as well as in immunological disorders. We aim to highlight the significant role of DMS technology in advancing the forefront of immunological research and inspiring deeper exploration in this field in the future.

## 2. Deep Mutational Scanning Methods and Process

**Figure 1:**
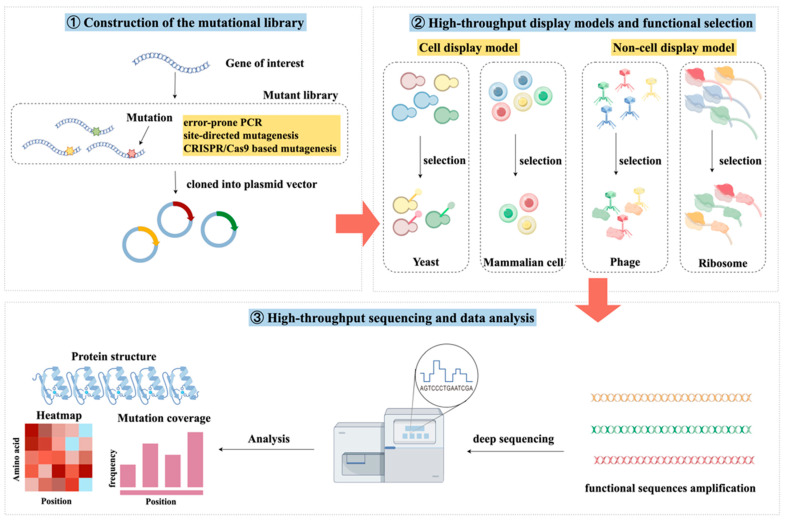
The workflow of deep mutational scanning. ➀ Saturation mutation is performed on the DNA codons at the target site of the target gene region, so that each position contains codons for all amino acids except the wild type, and each mutant sequence generated is collected to generate a mutation library. ➁ The mutant library is expressed based on a suitable protein display model (cell model or non-cell model), and functional variants are screened by giving specific functional selection (affinity or stability, etc.). ➂ The sequence information of the screened variants is obtained by high-throughput sequencing, and data visualization is achieved through a heatmap, etc. The data information generated based on DMS helps to establish the association between mutant sequences and protein functions.

DMS primarily comprises three main components: construction of mutant libraries, functional screening, and high-throughput sequencing analysis (Figure 1) [8]. The central concept is to link “site-variant-function” in a high-throughput framework: First, a mutant library containing many mutations is constructed using synthetic biology or directed mutagenesis, encompassing single-site or multi-site substitutions across defined regions or the full protein sequence. Second, an appropriate selection or screening strategy is applied to report on variant activity. In addition to molecular readouts such as binding affinity, catalytic activity and stability, functional phenotypes of cellular immune activities such as secretion, infection/entry, signaling pathway activation, and survival/proliferation can also be considered. Third, high-throughput sequencing quantifies variant abundances before and after selection to infer effect sizes and establish robust genotype–phenotype relationships [9]. This process allows for the stable mapping of site-level molecular effects to the cellular and even individual levels and can be combined with in vivo models for validation to obtain more physiologically relevant immunological conclusions.

### 2.1. Construction of the Mutational Library

Ideally, the DMS library encodes every possible amino acid substitution in the protein of interest. Therefore, the success of DMS depends on high coverage of mutant sequences, which requires the use of efficient DNA synthesis and cloning strategies to ensure inclusion of all desired mutations [10].

Mutations are typically introduced by primer synthesis followed by polymerase chain reaction (PCR) to integrate mutations into DNA products for the construction of subsequent libraries [9]. Additionally, oligonucleotides containing mutation fragments can also be used as templates for homologous recombination and introduced into target cells (such as yeast or mammalian cells) through electroporation, liposome-mediated transfection, or other methods. The mutations are integrated into the genome by replacing corresponding regions of the target gene in the cells through homologous recombination mechanisms [11]. Early libraries were often generated by error-prone PCR, which lowers polymerase fidelity to stochastically introduce mutations and rapidly diversify sequences. However, this approach yields uneven mutational spectra with pronounced biases, leading to gaps in substitution coverage and frequent loss-of-function variants. An ideal random mutagenesis scheme would sample all nucleotides uniformly and maximize amino acid diversity—particularly when substituting three consecutive nucleotides. Therefore, the mutation rate needs to strike a balance between maintaining clone uniqueness and maintaining function to achieve optimal results [12,13]. This limitation is particularly prominent in immunology because small structural regions, such as antibody complementarity-determining regions (CDRs), are highly complex and have delicate functions. A single amino acid substitution can significantly change the affinity and specificity of antigen binding. Accordingly, systematic and precise amino acid saturation mutagenesis is essential to elucidate sequence–structure–function relationships in immune proteins. To overcome the limitations of error-prone PCR, programmed allelic series (PALs) were subsequently developed. PALs use synthetic oligonucleotides with degenerate codons (e.g., NNN/NNS/NNK) at specific sites, which can systematically cover all amino acid substitutions, thereby significantly reducing the bias caused by error-prone PCR [4]. In immunological applications, PALs have enabled targeted modification of antibody CDRs, such as combining single-stranded DNA with lambda exonuclease for DNA shuffling and achieving full coverage mutagenesis of the Complementarity-Determining Region 3 (CDR3) through NNK codons, facilitating systematic identification of residues governing antigen recognition [14]. However, PALs still have problems with uneven amino acid distribution and a large number of stop codons. To address these issues, the trinucleotide cassette (T7 Trinuc) design proposed by Krumpe et al. can achieve an equiprobable distribution of amino acids at each site while avoiding the introduction of stop codons, thereby further enhancing the diversity and effectiveness of the library [15,16].

In site-directed mutagenesis, traditional cassette approaches build on Kunkel mutagenesis, but these are time-consuming and limited in efficiency. Based on this, Firnberg and Ostermeier developed PFunkel, which combines Kunkel mutagenesis with Pfu DNA polymerase to enable rapid site-directed mutagenesis on double-stranded plasmid templates—typically within a single day [17]. PFunkel has been used to construct mutant libraries of tumor necrosis factor (TNF), pertussis toxin, and cancer target trophoblast cell surface antigen 2 (TROP2) antibodies, supporting high-resolution epitope mapping [18]. Although PFunkel has greatly improved the efficiency of site-directed mutagenesis, its scalability in long genes or multi-site mutagenesis is still limited. To further address these problems, scalable and uniform nicking mutagenesis (SUNi) has been developed in recent years. SUNi achieves higher uniformity and coverage, significantly reducing wild-type residues, by implementing double nicking sites on the template, optimizing the annealing temperature of the flanking homology arms, and introducing a GC clamp at the 5‘ end. This approach achieves greater uniformity and coverage while significantly reducing wild type. Compared with PFunkel, SUNi not only maintains strong scalability for long fragments and multi-gene targets, but also significantly improves overall library quality and screening efficiency [19].

Beyond strategies relying on oligonucleotide synthesis and plasmid cloning, CRISPR/Cas9 provides a versatile approach for generating high-coverage variants in situ across the genome. The basic idea is to generate programmable cuts at the target locus using Cas9, and then use oligonucleotides or fragment donors to guide homology-directed repair (HDR), thereby completing site-by-site replacements or small insertions/deletions. This allows for barcoding to read diversity and track allelic series. Complementary to random or degenerate codon strategies, CRISPR-mediated saturation mutagenesis emphasizes functional mapping in situ post-transcriptional/post-translational contexts, helping to reduce spurious or biased phenotypic signals caused by overexpression and ectopic expression. Its technical limitations mainly include heterogeneous editing accessibility (PAM/sequence context dependence), differences in HDR efficiency, and potential unintended indel/splicing effects. Therefore, it is recommended to include editing spectrum and diversity monitoring (such as targeted sequencing to assess substitution/indel distribution and wild-type residues) during the library construction stage, set positive/negative sites and neutral sites as controls, and include “editability/editing efficiency” as a covariate in subsequent analyses to improve the robustness and comparability of effect estimates [20,21].

### 2.2. Functional Screening

DMS requires a model suitable for high-throughput assays to link genotype with phenotype. Typically, the DMS display medium includes cell models that express the gene through steps such as transfection or transduction; for example, in the case of non-cell models, variants were synthesized or expressed through in vitro transcription and translation systems or reconstructed in vitro translation systems (PURE system) [22,23]. Each modality has distinct advantages and use cases: Cellular models can not only be used to analyze the binding or stability of immune-related proteins, but also through integrating mammalian cells and engineered T cell platforms, support systematic screening of multi-level functions such as antibody post-translational modification, immune cell secretion, viral infection response, and TCR specificity remodeling, thereby providing key support for immunology research and the development of immunotherapy strategies. By contrast, non-cell models offer tightly controlled biochemical environments that minimize cellular confounders and are well suited for screening variants affecting binding affinity or other intrinsic biochemical activities [16] (Table 1).

The cell models most commonly employed in DMS screening include yeast and mammalian cells [22,24,25,26]. Yeast offers rapid growth and short doubling times, facilitating large-scale culture and high-throughput screening, thus promoting its widespread application in DMS [26]. In addition, yeast benefits from mature genetic manipulation techniques, with broad applications in transfection and gene editing. Traxlmayr et al. combined yeast display with high-temperature selection to construct a human Immunoglobulin G1 Fragment crystallizable (IgG1-Fc) region mutation library and together with high-throughput sequencing to analyze the effects of residues on IgG1 stability, mapped the “stability landscape” of the IgG1 CH3 domain and inform its structural and evolutionary constraints [26]. However, yeast is not suitable for some human membrane proteins with complex folding or requiring specific glycosylation modification, so DMS related to immune proteins is increasingly performed in mammalian cells, which can provide post-translational modifications (such as glycosylation, phosphorylation, and ubiquitination) more closely resembling physiological states. These modifications are crucial for immune protein function and disease-related phenotypes [22]. Therefore, they have become an important platform for antibody drug engineering and immune receptor research. For example, during the antibody optimization process, DMS can guide the evaluation and modification of single-chain variable fragments (scFv) or antigen-binding fragments (Fab), which can subsequently be converted them into full-length glycosylated IgG in mammalian cells to meet the optimization requirements of therapeutic antibodies in terms of affinity and immunogenicity [21]. In addition, CRISPR-engineered human T-cell platforms enable systematic functional screens. 437 single amino acid variants and 260,000 combinatorial variants of more than 30 TCRs were analyzed by DMS, revealing the differences between TCR binding and antigen activation, and finding that some TCRs can still be strongly activated by antigens even if they cannot be detected by peptide/major histocompatibility complex (pMHC) tetramer binding [27]. This finding highlights the limitations of relying solely on binding assays. In terms of TCR specificity modification, systematic saturation and combinatorial mutagenesis of the CDR3 region have been used to generate large-scale libraries, which were introduced into Jurkat TCRβ-/- cells via lentiviral transduction to establish T-cell libraries. Through tetramer staining and flow cytometric sorting, researchers isolated TCR variants capable of recognizing the original epitope, novel epitopes, or both, followed by high-throughput sequencing for detailed analysis. The results showed that systematic DMS of the CDR3 region could achieve specific remodeling among closely related peptides, providing an important basis for analyzing the mechanism of TCR-pMHC interaction and developing more effective immunotherapies [28].

Non-cell models can efficiently evaluate the affinity phenotypes of many protein mutations while avoiding the complexity and variability that cellular systems might introduce. Common non-cell models include phage and ribosome display [2,29,30]. Phage display is the most widely used non-cell models and has enabled the discovery of hundreds of antibodies for research, diagnostic and therapeutic applications, especially antibodies against challenging targets and antibodies with tailored binding properties [2,23]. Phage display can be used to create and evaluate libraries of up to 1 × 10^12^ clones, and high-abundance libraries are conducive to the discovery of high-affinity antibodies against antigens [16,29]. Schofield et al. reported a high-quality phage display library containing over a billion human antibodies, from which over 38,000 recombinant antibodies targeting 292 antigens were produced. They validated 7200 unique clones through specificity testing, sequence analysis, and various biochemical assays, highlighting the immense potential of whole-genome monoclonal antibody development [31]. In contrast to phage display, ribosome display does not require cloning or living cells, thus avoiding many limiting factors such as growth environment control. Therefore, it has the advantage in expressing toxic proteins or selection in hostile conditions, it also supports extremely high diversity (more than 1 × 10^12^). However, because RNA is susceptible to hydrolysis and nuclease degradation, limited molecular stability remains a principal drawback of the ribosome display system [16,30].

### 2.3. High-Throughput Sequencing and Data Analysis

Analyzing mutation libraries is a powerful strategy to understand the functional implications of variants, but the traditional first-generation Sanger sequencing identifies only a small number of mutants per batch, limiting downstream functional analyses. However, the rapid development and application of high-throughput sequencing have enabled the simultaneous profiling of hundreds of thousands of variants, efficiently enriching for advantageous mutations and establishing a foundation for DMS. Multiple next-generation sequencing (NGS) platforms, including Illumina, Nanopore, ABI/SOLiD, Polonator and Pacific Biosciences, support DMS by delivering billions of bases at low per-base cost [32,33,34].

At the data analysis level, DMS analysis usually relies on the calculation of amino acid variant enrichment ratios to identify key mutations that affect function. Early tools and frameworks, such as Enrich v1.0 and EMPIRIC v1.0 [35,36] compare variant frequencies before and after selection to infer relative fitness and provide basic statistical modeling and visualization for large datasets. However, the core of such methods is still based on the comparison of variant frequencies, and the results are easily affected by experimental conditions, sample processing, and sequencing bias. Especially in immunological research, this bias may lead to the neglect of low-frequency mutants, which often correspond to key immunological functions. For example, the SARS-CoV-2 receptor binding domain (RBD) mutation E484K was initially rare in DMS outputs, but was later confirmed to be a major antibody escape site [37]. Similarly, in TCR studies, certain low-frequency clones may be difficult to detect in peptide-MHC tetramer binding assays yet display strong immune responses in functional readouts [27,28]. These findings suggest that ignoring low-frequency mutants may lead to biased interpretations of immune response mechanisms or pathogen evolutionary trajectories, posing significant challenges to the analysis of results. Therefore, to improve reliability and accuracy, more advanced bioinformatics tools and computational models are necessary to address these limitations. On this basis, Fowler et al. developed Enrich2 v1.1.0, a comprehensive statistical model for analyzing DMS data applicable to datasets with any number of time points. Enrich2 is based on a random effects model with repeated results. It not only provides improved scoring methods to effectively reduce noise and detect small-effect mutations but also estimates mutation scores and standard errors to reflect sampling errors and experimental consistency. However, it relies more on normalization and good repeated design when the depth is very low or there are strong batch differences [36]. DiMSum v1.1.3 also relies on Poisson sequence count distributions and considers empirical variance to estimate errors. DiMSum differs by introducing specific additive and multiplicative modifications to handle empirical variance. By sharing empirical variance across variants, DiMSum is more robust to overdispersion and has a lower requirement for replicate numbers. However, when there is a large dispersion or insufficient barcode collapse, the error estimates may be conservative or introduce systematic biases. DiMSum demonstrates similar performance to Enrich2 on datasets with less pronounced overdispersion [38]. Apart from Enrich2 and DiMSum, Bloom et al. introduced dms_tools v1.0.1 as an analysis software that infers the preference of each codon for each amino acid from the given selection pressure or assesses the extent to which these preferences change under different selection pressures and has been shown to be more accurate in inference from simulated data than simply calculating the ratio of counts before and after selection. The intuitive visualizations created by this software aid in result interpretation, guiding protein engineering, understanding sequence–structure–function relationships, and providing insights for developing better evolutionary models for sequence analysis, but does not integrate three-dimensional structure priors or epistasis modeling in the native statistical framework [39]. In practice, dms_tools has been widely used in immunology, including the analysis of neutralizing antibody escape of HIV-1 envelope protein (Env) [40], the mapping of mutational functions of influenza virus hemagglutinin (HA) [41], and SARS-CoV-2 RBD immune escape studies [37]. These studies have demonstrated the important value of dms_tools in the analysis of viral immune escape and antigenic evolution. Similarly, all three methods are limited by the inherent issues of count data, namely sensitivity to PCR bias, sequencing depth, and batch variation. Consequently, they may underestimate low-frequency, functionally relevant substitutions in immunological applications, covering a wide range of loci, from antigenicity/immunogenicity (including escape) to affinity and specificity, signal transduction and conformational regulation, folding stability, and expression/secretion/localization. In practice, selection can be based on data conditions, such as prioritizing dms_tools for low depth, using Enrich2 for multiple time points, and selecting DiMSum for overdispersion. Threshold calibration and reproducibility testing should be performed in independent batches or cohorts. Furthermore, structural and evolutionary features should be incorporated as covariates into subsequent models, and strict control should be exercised for data exceeding model expectations [36,38,39].

## 3. Application of Deep Mutational Scanning in Immunology

The application of DMS in immunology focuses on three key molecules: antibodies, antigens and TCRs, which correspond to the core components of humoral immunity, pathogen escape, and cellular immunity, respectively. By systematically mapping mutational effects, DMS enables us to understand the interactions between these core molecules and their mechanisms of action in immune responses, from molecular mechanisms to integrated immune functions.

### 3.1. Antibody Engineering

Antibody engineering has become an important field in biomedical research and drug development, aiming to improve the biological and functional properties of antibody candidates to enhance clinical efficacy [42]. As a versatile platform, DMS has shown its unique advantages among many technologies and methods through protein mutagenesis and functional screening combined with deep sequencing and bioinformatics, providing the possibility of improving the affinity, specificity, and stability of antibodies [43,44]. In addition, DMS can accurately map the fine conformational epitopes targeted by a given antibody, providing a better understanding of the structural basis of its protective mechanisms, which can enhance preventive or therapeutic interventions for human diseases.

Monoclonal antibodies (mAbs) isolated from immune or synthetic libraries can be further optimized to enhance their therapeutic properties, with affinity to their homologous antigens being a key determinant of functional efficacy. Protein therapeutics with precise in vitro affinity tuning have also provided new insights due to the application of DMS. For example, Fujino et al. implemented a systematic affinity engineering strategy for the Fab fragment of an antibody against the tumor necrosis factor receptor (TNF-αR). They first performed single-site DMS across the six CDRs of the heavy chain (VH) and light chain (VL) to constructed a comprehensive library of single amino acid substitutions, then used ribosome display for functional screening to identify beneficial mutations that increased antigen binding. These single-point mutations were then combinatorially optimized, and a significant improvement in antibody affinity was achieved with only seven amino acid substitutions, reducing the dissociation constant (Kd) from 7.28 nM to 3.45 pM, an overall improvement of more than 2000 times. This result not only demonstrated the powerful ability of DMS in rapidly screening beneficial mutations, but also demonstrated its unique advantages in guiding the combination of multiple mutations and achieving fine optimization of antibody affinity [23]. Building on this foundation, subsequent studies have further utilized DMS to not only to identify advantageous substitutions, but also systematically map the comprehensive fitness landscape of antibody–antigen binding, thus expanding the application of DMS in antibody engineering. Forsyth et al. developed DMS for antibody CDRs, capable of systematically assessing the impact of every possible single-point amino acid substitution on antigen binding. This method utilizes a full-length IgG library containing over 1000 CDR point mutations, displayed in mammalian cells, and sorted based on antigen affinity using flow cytometry. High-throughput sequencing is then used to analyze the enrichment or depletion of different mutations, thereby mapping the functional landscape of high-affinity, low-affinity and neutral mutations. When applied to the humanized anti-EGFR antibody hu225 (the parent antibody of cetuximab), this method covered 1121 single-point substitutions at 59 CDR positions across VH and VL regions, yielding a nearly comprehensive fitness landscape for antibody–antigen binding. Most substitutions were neutral or deleterious, but 67-point mutations that significantly improved affinity were identified. These not only verified the existing structural and functional data, but also revealed new optimized residues. DMS thus provides a robust tool for systematically analyzing the antibody–antigen interface and guiding antibody engineering [24]. Beyond mAb affinity optimization, DMS also informs potential in bispecific antibody design. Given the promise of dual-targeting antibodies in enhancing efficacy, Koenig et al. used a vascular endothelial growth factor (VEGF)/angiopoietin 2 (Ang2) dual action Fab (DAF) as a model, performed systematic DMS on the CDRs, and combined it with phage display and high-throughput sequencing. They not only identified beneficial mutations that enhanced VEGF or Ang2 binding, but also revealed synergistic effects and key stability sites between different CDR residues. Through further combinatorial optimization, the 5A12 antibody, which had an initial affinity of approximately 5 nM, was modified into multiple variants with sub-nanomolar affinities for both antigens, with blocking effects comparable to high-affinity monospecific antibodies. This provides the first demonstration that DMS can achieve dual affinity maturation, thereby expanding the design concept of bispecific antibodies. In addition to showing application prospects in neovascular age-related macular degeneration, this strategy is also applicable to VEGF/Ang2-driven abnormal tumor vascular remodeling and immunosuppression, and is expected to improve the tumor immune microenvironment, enhance immune cell infiltration, and enhance anti-tumor effects [45]. In addition to optimizing affinity and specificity, DMS enables high-resolution conformational epitope mapping. Using yeast display coupled with DMS, Kowalsky et al. mapped the binding epitopes of infliximab and TNF, Hu1B7 and pertussis toxin, and confirmed that the conformational epitopes obtained by this method were highly consistent with existing data, demonstrating the reliability of the experimental process. Compared with traditional low-throughput methods that rely on co-crystallization or mass spectrometry, this new strategy combining comprehensive mutagenesis, cell surface display and deep sequencing can quickly map high-resolution epitope maps of multiple antibody–antigen complexes in a shorter time, at a lower cost and with reduced antibody consumption. These datasets not only revealed the functional contribution of single-point mutations in antigen binding, but also provided data support for predicting escape mutations, evaluating cross-reactivity, and improving protein–protein interaction computational models. Although this method still has certain limitations when dealing with antigens with complex structures or those that rely on glycosylation modifications, it has been successfully applied to complex proteins such as TNF and TROP2, showing its broad prospects in immunology research and antibody drug development [18]. Based on this, researchers have gradually realized that the potential of DMS is not limited to structural and functional elucidation but can also extend to clinically relevant engineering problems. For example, maintaining antibody binding activity while reducing its immunogenicity is a pressing challenge in antibody drug development. After antigen processing, antibody variable regions are cleaved into peptides of approximately 15 amino acids, which fit into the HLA-II binding groove with varying registers. Because CDRs (particularly CDRH3) are often enriched with aromatic and hydrophobic residues, these side chains physiochemically favorably match key pockets (typically P1, P4, P6, and P9) within the HLA-II groove. Consequently, high-affinity HLA-II epitopes are formed at the variable region-framework interface or within the CDRs, resulting in structural overlap with functional CDRs. On this basis, strategies to reduce HLA-II binding affinity include first identifying the registration and anchoring sites of the epitope, followed by site-specific substitutions with mismatched physiochemical properties. For example, aromatic or branched residues that fit the hydrophobic pocket can be replaced with charged or strongly hydrophilic residues, or perturbations can be introduced at key positions to alter registration, thereby weakening the peptide’s anchoring in the pocket, reducing binding free energy and stability, and thus diminishing presentation and T cell recognition. To minimize the impact on antigen binding function, such replacements should be preferentially placed on side chains or CDR edges or adjacent framework sites that are not directly involved in antigen binding, and verified using DMS functional readout [46,47]. To this end, Sivelle et al. proposed a strategy combining T cell epitope prediction with DMS to reduce the immunogenicity of therapeutic antibodies. Because CD4+ T cell epitopes often overlap with antibody CDRs, and direct removal is challenging. They first used the netMHCIIpan3.2 algorithm to predict HLA class II binding in the heavy chain complementarity-determining region 2 (HCDR2) and heavy chain complementarity-determining region 3 (HCDR3) regions, covering 46 alleles representing approximately 90% of the global population. Heatmap was used to identify permissive substitutions that could reduce HLA binding. DMS was then combined with yeast display to screen for mutations that both weakened human leukocyte antigen (HLA) binding and maintained antigen binding. Based on this information, combinatorial libraries were constructed to isolate functional clones. Using the anti-TNF-α antibody adalimumab as a model, the study identified approximately 200 mutants with lower HLA binding scores than the original antibody. When constructed as full-length antibodies, three of these mutants showed higher TNF-α affinity and neutralizing activity than adalimumab. These results indicate a degree of immunogenicity tolerance in antibody sequences and demonstrate that integrating DMS with epitope prediction can reduce immunogenicity, prolong in vivo efficacy, and mitigate anti-drug antibody (ADA) development while maintaining or even enhancing function [48].

### 3.2. Antigen Epitope Identification

Antigen epitope identification is a critical step in vaccine development, enabling precise characterization of how antibodies engage their antigenic targets. Unlike traditional approaches such as Enzyme-Linked Immunosorbent Assay (ELISA), enzymatic digestion, or chemical cleavage, which only offer limited sequence analysis, the high resolution of DMS can interrogate saturated mutational coverage of antigenic targets, thereby accurately identifying and analyzing epitopes to provide key escape mutation information. In recent years, DMS has been widely applied in virology, especially for rapidly evolving pathogens including SARS-CoV-2, influenza virus, and Zika virus. DMS can better understand how viruses evade the surveillance of the immune system and thus assist in designing effective vaccines, which is crucial for addressing the challenges posed by evolving viruses [49].

The RBD of the SARS-CoV-2 spike glycoprotein mediates viral attachment to the angiotensin converting enzyme 2 (ACE2), determining host range and serving as a dominant target of neutralizing antibodies [50,51,52]. Starr et al. utilized the yeast surface display platform to perform DMS on a library of mutations in the SARS-CoV-2 RBD region, investigating how these variants affect ACE2 binding affinity and protein expression. Although several substitutions increased ACE2 binding, these mutations did not exhibit a selective advantage in the circulating strains of SARS-CoV-2 [53]. The complex relationship between the biochemical phenotype of RBD presented by yeast and viral fitness, along with the method primarily focusing on measuring antibody binding limit its application scope [53]. By contrast, assays that evaluate neutralization using full-length spike in cellular infection models are considered more directly relevant to protection and better capture viral adaptability and immune responses [54]. Dadonaite et al. used non-replicative pseudotyped lentiviruses to create libraries of the Omicron BA.1 and Delta spikes, with DMS directly quantifying the impact of mutations on antibody neutralization. These measurements showed good correlation with traditional pseudovirus neutralization assays [54]. On this basis, they expanded the evaluation to over 9000 mutations in the XBB.1.5 and BA.2 spike proteins, analyzing multiple functions including ACE2 binding, cell entry, and escape from human serum. DMS analysis results revealed mutation sites on the spike protein that significantly affect ACE2 binding or enhance the ability to escape from the serum of breakthrough infected individuals [55]. These data show that DMS provides important insights into the evolution of SARS-CoV-2.

Similarly, mutation maps based on DMS have also been applied to reveal epitope escape information for other viruses, such as Zika virus (ZIKV) and influenza virus. Sourisseau et al. performed DMS to systematically saturate mutagenesis of the Zika virus E protein to generate high-resolution functional landscapes of viral growth and antigenicity [56]. The study not only revealed differences in mutation tolerance among different residues, such as disulfide bond cysteines and histidines associated with low-pH conformational transitions being highly sensitive to mutation, while surface residues were relatively tolerant, but also verified the consistency of these patterns with existing structural understanding. Further analysis showed that mutations in the fusion loop and key linker regions of domains I-III were strictly restricted, which are the core of viral receptor binding and membrane fusion. They also systematically mapped the antigenic escape mutations of two monoclonal antibodies and found that the selective pressure of neutralizing antibodies would promote the enrichment of escape mutations in the population generated by Vero cells [56,57,58]. These results not only validated the reliability of DMS within a single virus system, but also indicate its potential to reveal immune-escape patterns across diverse viruses. Therefore, DMS has been extended to the hemagglutinin (HA) protein of influenza virus. Using a modified DMS strategy for H1N1 strains, researchers systematically evaluated the functional effects of nearly all amino acid substitutions in the influenza A virus HA gene and achieving 98% coverage. By combining a large-scale mutant library with deep sequencing and employing a “helper virus” approach to generate the viral library, they effectively overcame the bottlenecks inherent in traditional plasmid construction, significantly improving the accuracy and reproducibility of the measurements. The resulting fitness maps illuminated the diverse contributions of HA residues to viral replication and immune function: antigenic sites in the globular head were highly tolerant to mutation, consistent with their propensity for immune escape. In contrast, the stem region targeted by broadly neutralizing antibodies, disulfide bond-forming cysteines, and key histidines involved in low-pH conformational changes were subject to strict mutational constraints, underscoring their potential as vaccine or therapeutic targets. Phylogenetic analyses further confirmed that these experimental results accurately reflected evolutionary constraints on HA, with most sites exhibiting conserved amino acid preferences across homologs, thereby supporting the robustness and generalizability of the approach. Moreover, by comparing in vitro and in vivo fitness, DMS can identify variants that achieve high production titers yet display attenuated phenotypes in the host, informing the design of live-attenuated vaccine candidates. Overall, DMS not only deepens our understanding of the HA structure–function relationship and immune escape mechanisms, but also provides important references for predicting influenza evolutionary pathways, building more accurate viral evolution models, and guiding the design of antiviral drugs and vaccines. It also highlights the broad applicability of DMS to other genetically tractable viruses and microbial genomes [59,60,61]. Lee et al. also revealed that favorable mutations are enriched in evolutionary successful lineages by measuring the effects of all single amino acid mutations in H3N2 strain HA on viral growth based on DMS. However, comparisons between H3 HA and H1 HA data showed significant differences in amino acid preferences and mutation tolerances, emphasizing the importance of experimental measurements in understanding viral evolution, although their effectiveness depends on the similarity between experimental and natural strains [62].

### 3.3. Recognition by T Cell Receptors

TCRs coordinate cellular immunity by recognizing short peptide antigens bound and presented by MHC molecules [63]. DMS has made significant advances in the TCR field, providing new strategies to enhance the affinity and specificity of TCRs for pMHC complexes. Screening of mutation libraries based on DMS has laid a solid foundation for the development of efficient and safe TCR-based immunotherapies.

TCR affinity is inherently low, making it a target for protein engineering to improve stability and affinity. Affinity maturation efforts typically concentrate on residues within the CDR loops at the binding interface [64]. Sharma et al. used DMS to comprehensively analyze the functional residues of TCRs in their recognition of the cancer antigen MART-1·HLA-A2. Unlike previous affinity maturation approaches that focused solely on CDRs, this study systematically included Vα/Vβ interfacial and framework residues for analysis, constructing a single-codon library covering both CDR and non-CDRs. The results revealed that in addition to CDR residues, the Vα/Vβ interface is critical for proper folding and stable binding. Notably, some interface mutations (e.g., F45βY) significantly enhanced TCR stability and affinity: affinity increased approximately 50-fold in yeast display and 60-fold in vitro assays, while maintaining high specificity for MART-1·HLA-A2. Further experimental validation using a two-codon interface library confirmed the potential of these interface residues for affinity and stability optimization. These results demonstrate that interface residues distal to the binding site can substantially modulate TCR function, supporting their inclusion in DMS and directed evolution strategies. This discovery not only broadens our understanding of the TCR structure–function relationship but also provides a viable optimization path and theoretical basis for soluble TCR engineering and adoptive T cell therapy [65]. In addition to the systematic exploration of interface residues, some studies have further combined DMS with directed evolution to map the sequence fitness landscape of TCR-peptide-MHC interactions more comprehensively and to propose principled optimization strategies. Harris et al. optimized the interactions of cancer antigen-specific RD1-MART1HIGH TCR with pMHC by prioritizing DMS enriched substitutions. Research demonstrates that combining directed evolution with DMS can comprehensively characterize the sequence fitness landscape of TCR-peptide-MHC interactions, overcoming limitations of traditional phage or yeast display approaches, which focus on point-by-point modifications within the CDRs. Using the cancer antigen-specific TCR RD1-MART1HIGH as an example, DMS systematically screened for beneficial mutations at both interface and non-interface residues. These substitutions were found not only to enhance binding individually but also to markedly enhance TCR affinity and cell-surface expression when combined. Results showed that affinity could be increased by over 200-fold and yeast surface expression by approximately 6-fold. The study also compared the fitness landscape-informed mutation combination strategy with a multi-codon library screening approach and corroborated this strategy with computational modeling. While computational simulations performed well in predicting mutations at the binding interface, they struggled to accurately predict affinity changes for mutations distal to the interface, highlighting the role of DMS data in complementing and calibrating computational models. Overall, this study demonstrates that DMS can not only efficiently identify and combine affinity mutations, but also take stability factors into account, providing strong experimental and theoretical support for TCR optimization design, cancer immunotherapy and soluble TCR engineering [66].

However, increasing TCR affinity usually does not improve its specificity, and the development of TCRs as immunotherapeutic agents is hindered by low specificity (intrinsic TCR cross-reactivity). Mechanistically, TCR recognition of pMHC is degenerate and relies on the plastic CDR3 loops to achieve induced fit, so a single TCR can accommodate multiple pMHCs with similar physicochemical features. Even when not located at the binding interface, engineered mutations can, via long-range allostery from the framework, alter CDR3 pre-organization and flexibility and indirectly rearrange interfacial interactions (hydrogen bonds/water bridges, salt bridges, hydrophobic packing, and aromatic stacking/cation–π), thereby redistributing binding energy and rewriting the energetic landscape of recognizable peptides. These structural changes manifest kinetically as shifts in k_on/k_off and K_D, with small decreases in k_off being amplified by kinetic proofreading to cross activation thresholds. When mutations restrict CDR3 conformational sampling and increase the k_off for off-target ligands, the recognition repertoire narrows and specificity improves; conversely, if mutations lower the off-target k_off or increase CDR3 flexibility, selectivity may relax and cross-reactivity rise. Thus, fine allosteric tuning of CDR3 pre-organization and dissociation kinetics—without directly altering the interface—is a key route to remodel the TCR recognition landscape [67,68,69]. In clinical applications, affinity-specificity is directly related to immune safety: increased affinity is often accompanied by increased cross-reactivity, and the risks are mainly manifested as on-target/off-tumor (low-level target antigens in normal tissues are attacked) and off-target/cross-reactivity (host peptides like the target peptide are used as targets). Especially when the CD8-independent activation threshold is obtained, it is more likely to be amplified. The consequences can be upgraded from general adverse reactions to lethal toxicity, including organ damage caused by misidentification of myocardial, neural or endocrine-related autologous peptides, systemic toxicity caused by attack of widely low-expressed targets, and cytokine storm and neurotoxicity induced by non-specific strong activation [63,67,68,69]. Various protein engineering strategies have been explored to enhance TCR specificity; thus, designing more specific TCRs has proven to be challenging [70,71,72]. Rosenberg et al. investigated the 868 TCR specific for the HLA-A2 presented HIV SL9 epitope and used DMS to assess whether framework mutations distal from the binding interface could modulate specificity. Single-site mutation libraries of α and β chains were generated using a yeast display system and these libraries covered all 6 CDR loops and adjacent framework regions. DMS analysis revealed that substitutions above the CDR3β loop did not significantly affect SL9 binding affinity but weakened recognition of escape variants, while mutations near the tip of CDR3α reduced the adaptability of TCR to different ligands. These findings indicate that non-interface framework mutations can enhance TCR specificity without changing binding affinity or introducing novel reactivities via direct interface alterations [63]. Thus, systematic mutation analysis combined with DMS provides a new approach to resolving the contradiction between TCR affinity and specificity optimization. However, improving affinity is often accompanied by increased cross-reactivity, which may lead to unexpected recognition of non-target antigens or host peptides by TCR, thereby causing serious side effects or even fatal reactions. Mitigating off-target recognition while improving efficacy and ensuring immune safety remains a central challenge for future TCR engineering.

## 4. Conclusions and Future Perspectives

As a rapidly advancing technology, DMS has introduced substantial innovations to immunology research. This review not only systematically introduces the core principles and experimental workflow of DMS, but also comprehensively analyzes its advantages and limitations in library construction, display platforms, screening models, and data analysis. Compared to traditional systematic point mutagenesis or directed mutagenesis, DMS achieves high mutation coverage and analytical depth by coupling saturation mutagenesis with NGS. Firstly, it can generate nearly all possible amino acid substitutions across the entire protein, thus overcoming the limitations of previously targeted modifications limited to individual sites. Secondly, NGS offers the ability to simultaneously analyze millions of variants, far exceeding the throughput and resolution limitations of Sanger sequencing. More importantly, DMS has demonstrated distinctive value in multiple core areas of immunology. In antibody engineering, it can systematically map the functional landscape of antibody CDRs, identify key residues that improve affinity and stability, and guide the optimization of multiple mutational combinations. In epitope mapping, DMS provides high-resolution antigen escape maps, revealing the fine structural features of conformational epitopes and providing predictive evidence for vaccine development. In TCR research, DMS has transcended the limitations of focusing solely on CDRs, expanding its analysis to include framework regions and the Vα/Vβ interface, enabling a systematic analysis of TCR-peptide/MHC interactions. These applications not only deepen our understanding of the structure–function relationship between antibodies and receptors but also provide strong technical support for optimizing immunotherapy strategies, modifying antibody drugs, and predicting immune escape, demonstrating its enormous potential to advance immunology research and applications.

### 4.1. Challenges and Limitations

Despite the importance of DMS in immunology, the interpretation and generalization of DMS findings to actual immune responses or clinical applications remain limited. First, regarding experimental systems, most available DMS datasets are derived from living cell environments, including mammalian cells, yeast, and bacterial models. These platforms enable the expression of human or pathogenic proteins under conditions more closely resembling physiological conditions, thus more realistically reflecting structure–function relationships. These methods offer advantages in terms of high throughput and rapid analysis of relatively simplified biochemical phenotypes, such as protein-ligand binding, but their ability to characterize complex cellular processes and upstream and downstream signaling is limited. It is important to emphasize that even DMS performed in living cell systems, such as mammalian cells, cannot fully recapitulate the complexity of the in vivo immune microenvironment. Gradients of antibodies and cytokines across tissues, the spatial organization, and interactions among immune cells, as well as dynamic regulation driven by inflammation, infection, and metabolic states, all influence the true outcomes of antigen–antibody binding or receptor-ligand interactions. Such differences may result in functional effects observed in experiments diverging from those occurring in vivo, thereby constraining the translational potential and predictive accuracy of DMS data.

Secondly, DMS relies heavily on a designable, quantifiable functional screening readout. This means that DMS is most effective when the biological function of the target protein is well-defined and can be operationalized as quantifiable, high-throughput screening metrics (such as binding, secretion, infection, signaling activation, and cell growth/survival). By contrast, scalable, generalizable screening paradigms remain scarce for immune molecules with poorly characterized functions or those dependent on multicellular circuits or tissue structures. The overall physiological effects of such molecules (such as immune response integration, metabolic regulation, and neuro-immune coupling) are difficult to quantify within existing standardized DMS workflows, limiting the potential for systematic research. When quantifiable phenotypes are limited, dependence on library quality and sampling balance is further magnified. On this basis, library representativeness and coverage also pose practical constraints: oligonucleotide synthesis bias and bottlenecks in cloning and expression/viability lead to uneven sampling, reducing the actual proportion of certain amino acid substitutions and their combinations in the dataset, thereby undermining the stability of effect estimates and tending to systematically underestimate epistasis as well as regions constrained by post-translational modifications. Likewise, data analysis faces stability challenges. Because DMS relies on sequencing-based count readouts, it is highly sensitive to PCR amplification and sequencing depth, and batch effects are difficult to eliminate. Different normalization strategies and choices of hierarchical or mixed-effects modeling can also introduce substantial discrepancies, diminishing cross-platform and cross-study comparability and reproducibility, and further amplifying uncertainty in threshold setting (e.g., positive/negative, or pathogenic/benign) and evidence grading when extrapolating to clinical contexts [36,73].

Thirdly, existing evidence remains insufficient regarding the critical issue of the immune microenvironment and in vivo complexity. While DMS applications for antibodies, antigens, and TCRs are relatively mature, DMS work targeting immune checkpoints or complex receptor networks that can be functionally engineered and validated under conditions resembling the in vivo microenvironment remains limited. At present, only a few related explorations can provide indirect inspiration. For example, the structural study of the programmed cell death protein-1 (PD-1)/PD-L2 high-affinity complex provides residue-level information to support the development of small molecule immune checkpoint drugs, but it has not been fully integrated with the systematic mutation-function mapping of DMS [74]. these observations underscore the urgent need to extend DMS into research systems that more closely approximate in vivo immune complexity.

Finally, translational applications for clinical diagnosis and pathogenic mechanism elucidation are still in early stages, and systematic practices for typing diagnosis of immune diseases, grading of functional evidence of pathogenic variants, and even supporting personalized treatment decisions are still limited. Although current explorations have mostly focused on cross-analysis combining DMS data with population genetic variation databases (such as ClinVar and gnomAD) to determine whether a specific mutation is potentially pathogenic. For example, in the fields of cancer genetics and genetic diseases, several studies have linked DMS functional scores to clinical databases for variant reclassification and evidence grading. Taking breast cancer gene 1 (BRCA1) as an example, functional scores derived from saturated gene editing correlate well with pathogenic/benign annotations in ClinVar and provide actionable evidence for reclassification of variants of uncertain significance (VUS). Similarly, DMS readouts of transcriptional activation in tumor protein p53 (TP53) can distinguish between clinically pathogenic and benign alleles, and functional scores for phosphatase and tensin homolog (PTEN) are also consistent with clinical phenotypes and database annotations. This practice demonstrates a relatively general workflow: accurately mapping the site-variant-effect size matrix generated by DMS to variant coordinates in ClinVar/gnomAD, removing low-depth or low-confidence sites, calculating consistency metrics between the scores and clinical labels, and performing threshold calibration and reproducibility testing in independent cohorts or prospective samples [75,76,77,78,79,80,81,82,83]. However, the overall process is still at the stage of methodological validation and small-scale application, especially in autoimmune disease-related research. It should be noted that in the overall field of clinical diseases, systematic cases of immune-related diseases are still relatively scarce. On the one hand, the pathological phenotypes of many immune molecules require the coordination of multicellular circuits and tissue structures to be manifest, and it is difficult to obtain stable and quantifiable DMS readouts in the short term. On the other hand, there is a lack of prospective validation cohorts that are directly aligned with clinical outcomes (diagnosis, disease subtype, efficacy, or prognosis), resulting in insufficient calibration of thresholds and strength of evidence (Table 2).

Overall, the barriers limiting the clinical translation of DMS primarily include the gap between experimental settings and the in vivo immune microenvironment: in vitro and heterologous systems cannot recapitulate tissue-level antibody/cytokine gradients and cell–cell interactions, thereby reducing the reliability of clinical extrapolation. At the same time, DMS readouts depend on clearly defined, quantifiable phenotypes, which makes it difficult to cover complex immune functions that manifest only through multicellular circuits and tissue-level coordination. Furthermore, library representativeness and coverage are constrained by bottlenecks in oligonucleotide synthesis, cloning, and expression/viability, leading to uneven sampling and a tendency to underestimate epistasis and regions constrained by post-translational modifications. On the analysis side, sequencing-based count data are sensitive to batch effects and modeling choices, which weakens cross-platform comparability and makes threshold setting and evidence grading for clinical extrapolation less stable. In addition, functional engineering and validation under more physiologically relevant conditions remain insufficient, and clinical applications are largely at the stage of methodological validation and small-scale pilot use. Consequently, DMS is presently better suited to provide mechanistic and supportive evidence; its clinical interpretability will continue to depend on calibration and validation within near-physiological models and more robust analytical frameworks.

### 4.2. Future Perspectives

Despite the limitations, DMS holds great promise in immunology. Looking ahead, the key lies in translating the “site-variant-function” readout at the cellular level and mapping it to immune outcomes at the tissue and individual levels. To this end, DMS can be deeply coupled with single-cell multi-omics to simultaneously analyze cell states, lineage relationships, and functional outputs within the same mutational context. In the complex immune system, numerous correlations often exist, but causal relationships are difficult to directly confirm. By combining CRISPR-Cas9 and DMS technologies, precise, controllable perturbations at key nodes of immune pathways are expected to more clearly determine whether changes in specific molecules directly lead to downstream immune responses, thereby substantially improving the reliability of causal inferences [85,86,87,88,89,90]. Furthermore, by utilizing phenotype-barcode and time-series sampling, longitudinal selection pressure tracking can be performed under conditions such as cytokine secretion, pathogen infection, or drug pressure, thereby characterizing the dynamic trajectory of mutation effects as the microenvironment changes. Organoid and tissue slice co-culture models enable readouts of complex phenotypes such as secretion, migration, chemotaxis, and cell interactions in contexts that more closely recapitulate in vivo spatial structure, cell composition and matrix composition. In vivo platforms such as humanized mice can help connect the above readouts to endpoint indicators of tissue remodeling and clinical patterns, thereby enhancing the physiological relevance and inferential value of DMS results [91,92,93,94,95,96,97]. At the data and model level, multimodal integration of DMS count data with proteomics, transcriptomics, and other data can improve the detection and interpretability of low-frequency but critical mutations using hierarchical/mixed effects models and causal inference methods [98,99,100]. At the same time, structural biology and computational modeling should be incorporated the effects of mutations on three-dimensional conformation, binding interface, and homeostasis into the analytical framework. Combined with machine learning and deep learning, variants with better stability, affinity, and specificity can be pre-prioritized during the in vitro design stage [101]. At the translational level, the combination of all the above strategies is expected to elevate the cell-level readout of DMS to evidence that can be used for clinical interpretation, including functional annotation and classification of immune-related variants, to support the classification, diagnosis, and therapeutic stratification of immune diseases. This will also facilitate the optimization of antibodies, receptors, and ligands, providing a quantitative basis for target and site identification in diagnostics. Furthermore, by linking patient samples (such as peripheral blood mononuclear cells (PBMCs) and tumor-infiltrating immune cells), a functional evaluation pipeline aligned with clinical efficacy endpoints will be established, enabling the design and optimization of personalized treatment plans. Overall, through technological integration and methodological innovation, DMS will provide more precise and comprehensive tools for immunology research, advancing our understanding and clinical utilization of the immune system.

## Figures and Tables

**Table 1:** Display platform for DMS.

Display Type	Characteristic	Advantages	Limitation	Refs.
Yeast display	The target fragment (such as an antibody fragment, receptor or antigen mutant) is anchored and fused to the yeast cell surface so that it is fixed on the yeast surface for display.	Eukaryotic system and capable of some post-translational modifications Proven gene manipulation and library construction methods Suitable for large-scale mutation library screening	Human proteins are not suitable for complex folding or require specific glycosylation	[22]
Mammalian display	Rely on viral or plasmid vectors to introduce mutants into cells one by one, and display them inside cells or on the surface through transmembrane anchoring or secretion capture.	Closest to physiological conditions Retains intact post-translational modifications (glycosylation, phosphorylation, etc.) Suitable for antibody drug and immune receptor research	High cost Lower throughput than yeast/phage, limited library size (typically ≤1 × 10^6^)	[16,23,24]
Phage display	The target protein (usually an antibody fragment) is fused and expressed on the phage coat protein, and screening is achieved through phage proliferation and selection.	Large library size (1 × 10^9^–1 × 10^12^) Low cost and high throughput Widely used in antibody discovery	Lack of eukaryotic post-translational modifications Some proteins fold inefficiently	[16]
Ribosome display	Generating ribosome-mRNA-protein complexes through stop-codon-free translation enables genotype-phenotype coupling, which is then screened through ligand binding and high-throughput sequencing.	No cloning or cell culture required Library size can reach >1 × 10^12^ Suitable for proteins that are toxic or difficult to express in cells	RNA is prone to degradation and the system exhibits limited stability Lacks post-translational modifications	[16]

**Table 2:** Current Challenges and Potential Solutions for DMS.

Application	Challenge	Potential Solutions	Refs.
Antibody optimization	Some mutations may significantly improve antigen binding (high affinity), but may also disrupt antibody expression or structure, making it difficult to assess in vivo function.	Combine structural modeling to predict conformational stability and verify antibody function through organoid models or other in vivo experiments.	[16,42]
Antigen escape	Epitope regions are complex and constantly mutate under host immune pressure. A single cell-based or cell-free screening platform may not truly reflect the infection process (it cannot simulate the interaction between immune cells and pathways).	Design multi-site combination mutations and verify escape variants in combination with organoid or animal models.	[49,53,82]
TCR recognition	TCR–MHC interactions are highly dependent on MHC context and peptide conformation.	Designing multi-MHC parallel DMS, combining structural simulation and functional screening.	[63]
Complex or poorly defined immune molecules	Unable to build a function-dependent screening system, making it difficult to quantify mutation function through high-throughput.	Joint proteome/transcriptome prediction functional modules.	[11,84]
Clinical diagnosis	Lack of large-scale immunology database combined with DMS research, the immune system is highly personalized.	Combined database cross-analysis.	[81]

## Data Availability

No new data were created or analyzed in this study. Data sharing is not applicable to this review.

## Abbreviations

The following abbreviations are used in this manuscript:

ACE2	angiotensin converting enzyme 2
ADA	anti-drug antibodies
Ang2	angiopoietin 2
CDR	complementarity determining region
DAF	dual action Fab
DMS	deep mutational scanning
ELISA	enzyme-linked immunosorbent assay
Fab	antigen-binding fragments
HCDR	heavy chain complementarity-determining region
HDR	homology-directed repair
HLA	human leukocyte antigen
IgG1	immunoglobulin G1
mAbs	monoclonal antibodies
MHC	major histocompatibility complex
PALs	programmed allelic series
PCR	polymerase chain reaction
PD-1	programmed cell death protein-1
RBD	receptor binding domain
scFv	single-chain variable fragments
SUNi	scalable and uniform nicking mutagenesis
TCR	T-cell receptor
TROP2	trophoblast cell surface antigen 2
TNF	tumor necrosis factor
TP53	tumor protein p53
PBMC	peripheral blood mononuclear cell
PTEN	phosphatase and tensin homolog
VEGF	vascular endothelial growth factor
VUS	variants of uncertain significance
